# Intrapallidal injection of cannabidiol or a selective GPR55 antagonist decreases motor asymmetry and improves fine motor skills in hemiparkinsonian rats

**DOI:** 10.3389/fphar.2022.945836

**Published:** 2022-09-02

**Authors:** Felipe Patricio, Eliud Morales Dávila, Aleidy Patricio-Martínez, Nayeli Arana Del Carmen, Isabel Martínez, José Aguilera, Jose Manuel Perez-Aguilar, Ilhuicamina Daniel Limón

**Affiliations:** ^1^ Laboratorio de Neurofarmacología, Facultad de Ciencias Químicas, Benemérita Universidad Autónoma de Puebla, Puebla, Mexico; ^2^ Facultad de Ciencias Químicas, Benemérita Universidad Autónoma de Puebla, Puebla, Mexico; ^3^ Facultad de Ciencias Biológicas, Benemérita Universidad Autónoma de Puebla, Puebla, Mexico; ^4^ Laboratorio de Neuroquímica, Facultad de Ciencias Químicas, Benemérita Universidad Autónoma de Puebla, Puebla, Mexico; ^5^ Departament de Bioquímica i de Biologia Molecular, Facultad de Medicina, Institut de Neurociències, Universitat Autònoma de Barcelona, Barcelona, Spain; ^6^ Centro de Investigación Biomédica en Red sobre Enfermedades Neurodegenerativas (CIBERNED), Barcelona, Spain

**Keywords:** cannabidiol, CID16020046, GPR55 receptor, behavioral test, motor asymmetry, fine motor skills, hemiparkinsonian rats, Parkinson's disease

## Abstract

Cannabidiol (CBD) presents antiparkinsonian properties and neuromodulatory effects, possibly due to the pleiotropic activity caused at multiple molecular targets. Recently, the GPR55 receptor has emerged as a molecular target of CBD. Interestingly, GPR55 mRNA is expressed in the *external globus pallidus* (GPe) and striatum, hence, it has been suggested that its activity is linked to motor dysfunction in Parkinson’s disease (PD). The present study aimed to evaluate the effect of the intrapallidal injection of both CBD and a selective GPR55 antagonist (CID16020046) on motor asymmetry, fine motor skills, and GAD-67 expression in hemiparkinsonian rats. The hemiparkinsonian animal model applied involved the induction of a lesion in male Wistar rats *via* the infusion of the neurotoxin 6-hydroxydopamine (6-OHDA) into the medial forebrain bundle *via* stereotaxic surgery. After a period of twenty days, a second surgical procedure was performed to implant a guide cannula into the GPe. Seven days later, lysophosphatidylinositol (LPI), CBD, or CID16020046 were injected once a day for three consecutive days (from the 28th to the 30th day post-lesion). Amphetamine-induced turning behavior was evaluated on the 14th and 30th days post-injury. The staircase test and fine motor skills were evaluated as follows: the rats were subject to a ten-day training period prior to the 6-OHDA injury; from the 15th to the 19th days post-lesion, the motor skills alterations were evaluated under basal conditions; and, from the 28th to the 30th day post-lesion, the pharmacological effects of the drugs administered were evaluated. The results obtained show that the administration of LPI or CBD generated lower levels of motor asymmetry in the turning behavior of hemiparkinsonian rats. It was also found that the injection of CBD or CID16020046, but not LPI, in the hemiparkinsonian rats generated significantly superior performance in the staircase test, in terms of the use of the forelimb contralateral to the 6-OHDA-induced lesion, when evaluated from the 28th to the 30th day post-lesion. Similar results were also observed for superior fine motor skills performance for pronation, grasp, and supination. Finally, the immunoreactivity levels were found to decrease for the GAD-67 enzyme in the striatum and the ipsilateral GPe of the rats injected with CBD and CID16020046, in contrast with those lesioned with 6-OHDA. The results obtained suggest that the inhibitory effects of CBD and CID16020046 on GPR55 in the GPe could be related to GABAergic overactivation in hemiparkinsonism, thus opening new perspectives to explain, at a cellular level, the reversal of the motor impairment observed in PD models.

## 1 Introduction

Cannabidiol (CBD) is the second most abundant component of *Cannabis sativa* L., after Δ^9^-tetrahydrocannabinol (THC), which is responsible for the psychoactive properties of plant (Pertwee, 2008; Ibeas Bih et al., 2015; Pellati et al., 2018). As CBD does not directly activate the cannabinoid receptor type 1 (CB_1_), it is, thus, devoid of the psychoactive side effects exhibited by THC and is also considered a safe drug (Pisanti et al., 2017). Given its action as a pleiotropic molecule and its targeting of various proteins of the cannabinoid system, CBD can be described as a neuromodulator, with an increasing amount of data evidencing its neuromodulatory properties in different neurological and neuropsychiatric disorders (Fernández-Ruiz et al., 2013; Campos et al., 2017; Patricio et al., 2020; Xiong and Lim, 2021). Moreover, cannabinoid-related receptors, such as the G-protein coupled receptor 55 (GPR55), may play an important role in the effects exerted by CBD in the central nervous system (CNS) (Ryberg et al., 2007; Ligresti et al., 2016; Kaplan et al., 2017).

It has been shown that GPR55 is coupled to the proteins G*α*_12/13_ and G*α*_q_, which are activated by cannabinoids and the endogenous ligand of the GPR55, lysophosphatidylinositol (LPI); therefore, it promotes both the Rho-associated protein kinase (ROCK) and phospholipase C (PLC) pathways (Henstridge et al., 2011; Alhouayek et al., 2018). The cellular effects of the activation of GPR55 are the release of intracellular calcium from the endoplasmic reticulum (ER), the phosphorylation of the protein ERK1/2, and the activation of ROCK (Oka et al., 2007; Ryberg et al., 2007; Lauckner et al., 2008). Interestingly, data shows that CBD acts as an inverse agonist of GPR55 and, therefore, inhibits the LPI-induced stimulation of ERK1/2 phosphorylation (Anavi-Goffer et al., 2012) and the stimulation of [^35^S]GTPγS binding by LPI (Ford et al., 2010). Research has revealed the structure-activity relationship of GPR55 with the selective antagonist CID16020046, while the antagonism of GPR55 has been proposed as an efficacious treatment for several neurological diseases (Brown et al., 2018). CID16020046 has been used to study the physiological role of GPR55 in nociception (Okine et al., 2020), cognitive processes (Marichal-Cancino et al., 2016; Hurst et al., 2017; Marichal-Cancino et al., 2018), and the neuroprotective effects observed in neuroinflammation and Dravet syndrome animal models (Kaplan et al., 2017; Shen et al., 2022).

Early reports have demonstrated a high level of GPR55 mRNA expression in different areas of the CNS in both humans (Sawzdargo et al., 1999; Wu et al., 2013; García-Gutiérrez et al., 2018; Hill et al., 2018) and rodents (Ryberg et al., 2007; Sylantyev et al., 2013; Wu et al., 2013). As the main regions in which this expression has been observed are the striatum, hippocampus, cortex, and brainstem, it has been related to various behavioral processes, such as learning, memory, food intake, the emotions, and movement control (Marichal-Cancino et al., 2017). Moreover, impaired movement coordination in GPR55 knockout mice has been observed, thus suggesting that the receptor plays a role in motor control, given the relationship between its presence in the striatum and its potential link to the dopaminergic system (Wu et al., 2013). Indeed, PD is a neurodegenerative disorder characterized by dopamine deprivation in various nuclei of the basal ganglia (BG) due to dopaminergic neuron loss in the *substantia nigra pars compacta* (SNpc) (Smith and Villalba, 2008; Rommelfanger and Wichmann, 2010; Benazzouz et al., 2014).

The BG is an interconnected group of subcortical nuclei, in the deep encephalon, which, in a healthy state, are responsible for movement planning and modulation (Lanciego et al., 2012). The functional disruption of the BG causes the manifestation of motor symptoms such as akinesia, muscle rigidity, and tremor at rest (Rommelfanger and Wichmann, 2010; Galvan et al., 2015; Neumann et al., 2018). According to the classic BG model (Albin et al., 1989), the *external globus pallidus* (GPe) has been considered a relevant factor in PD, as it is highly over-inhibited in parkinsonism due to the loss of striatal dopaminergic activity leading to the disinhibition of the D_2_-striatal projection neurons. These processes are attributed to the hypokinetic states observed in the PD, in both in animal models and human patients (Berardelli et al., 2001; Galvan and Wichmann, 2008; Dong et al., 2021). Interestingly, the GPR55 mRNA transcript has been identified, *via in situ* hybridization, in the GPe and striatum of mice, as well as other nuclei of the BG circuit that are associated with movement control (Celorrio et al., 2017).

Recent studies have reported that GPR55 may serve as a therapeutic agent in the treatment of PD. Celorrio et al. (2017) studied the effect of the peripheral administration of abnormal cannabidiol (a synthetic isomer of CBD), to explore its agonist effects on the GPR55 receptor. They observed a beneficial neuroprotective effect on dopaminergic neurons injured by 1-methyl-4-phenyl-1,2,3,6-tetrahydropyridine (MPTP) and an anti-inflammatory and anti-cataleptic effect as a result of GPR55 activation in parkinsonian mice models. Burgaz et al. (2021) found similar results in two PD models, which revealed the neuroprotective effect of VCE-006.1, which is a chromenopyrazole derivative with biased orthosteric and positive allosteric modulator effects on GPR55. They found that the drug reversed motor dysfunction, the loss of tyrosine hydroxylase-containing neurons, and the elevated glial reactivity detected in the SNpc of parkinsonian mice. Another study found that neurons expressing heteromers (CB_1_/GPR55) are more resistant to 1-methyl-4-phenylpyridinium-induced (MPP^+^) cell death (Martínez-Pinilla et al., 2019). These findings suggest the use of GPR55 as a potential neuroprotective agent and a therapeutic target for the treatment of PD. The role played by GPR55 in the striatum of hemiparkinsonian rats has been subject to recent research, which reported similar effects on locomotor activity, in terms of both agonism and antagonism of GPR55. It may be, therefore, that this receptor has a modulatory effect on motor behavior (Fatemi et al., 2021). The foregoing evidence suggests that GPR55 may play a role in the modulation occurring in several nuclei of the BG circuit and may have an impact on movement control in an animal model of PD.

The study of the hemiparkinsonian model in rodents has frequently used the neurotoxin 6-hydroxydopamine (6-OHDA) to induce dopaminergic neuronal death in the SNpc (Deumens et al., 2002; Francardo et al., 2017; Hernandez-Baltazar et al., 2017; Segura-Aguilar, 2018). Lesions induced *via* 6-OHDA in the medial forebrain bundle show generalized degeneration and, thus, dopaminergic denervation in both the striatum and GPe (Mallet et al., 2008; Kita and Kita, 2011; Fernández-Suárez et al., 2012; Mallet et al., 2012; Abedi et al., 2013; Dong et al., 2021). This model enables the assessment of quantifiable turning behavior, which can be correlated to the extent of the lesion, while it has also been shown that the 6-OHDA lesion model impairs both gross and fine motor skills (Galvan et al., 2001; Klein et al., 2007; Rattka et al., 2016). To date, the effects of either the administration of CBD or the selective antagonism of GPR55 in the GPe on the asymmetrical motor and fine motor skills of hemiparkinsonian rats are unknown. Therefore, the present study aimed to examine the effect of the intrapallidal injection of LPI, CBD, or CID16020046 and its repercussions on the motor behavior of animal models of PD unilaterally induced *via* 6-OHDA lesion.

## 2 Material and methods

### 2.1 Animals

Eighty-seven male Wistar rats, weighing 250–300 g at the start of the study, were obtained from the Claude Bernard Vivarium at the Benemérita Universidad Autónoma de Puebla (BUAP or Meritorious Autonomous University of Puebla) and individually housed in groups of five to six with water and food *ad libitum* in a temperature-controlled room (22 ± 2°C) under controlled light conditions (12:12-h light-dark cycle). All the procedures described in the present study were conducted in accordance with the *Guide for Care and Use of Laboratory Animals* of the Mexican Council for Animal Care (Official Mexican Standard NOM-062-ZOO-1999) and were approved by the BUAP’s Use of Laboratory Animals and Ethics Committee, under protocol number VIEP.buap/#2122/2017.

### 2.2 Stereotaxic surgery

#### 2.2.1 Unilateral dopaminergic lesion

The rats were anesthetized, *via* intraperitoneal injection, with a mixture of ketamine (70 mg/kg) and xylazine (10 mg/kg), and placed in a Stoelting stereotaxic apparatus (ITEM: 51,600, Wood Dale, IL, EE. UU.). The rat’s skull was then exposed and trepanned, with a microsyringe then introduced into the medial forebrain bundle (MFB) in order to administer a unilateral injection of 6-hydroxydopamine (6-OHDA) solution (16 µg/2 µL of saline containing 0.1% ascorbic acid), while the control group was administered 2 µL of vehicle without 6-OHDA. The stereotaxic coordinates used were: AP, -2.1 mm from the Bregma; ML, +2.5 mm from the midline; and, DV, -7.4 below the dura (Paxinos and Watson, 1998). Each solution was injected using a 10 µL Hamilton syringe coupled with a motorized injector (Nanomite Syringe Pump, Harvard Apparatus), at an infusion rate of 0.2 μL/min. For this part of the study, all animals were randomly divided into two groups: 6-OHDA (*n* = 47); and, vehicle (ascorbic acid) (*n* = 40).

#### 2.2.2 Guide cannula placement

**FIGURE 1:**
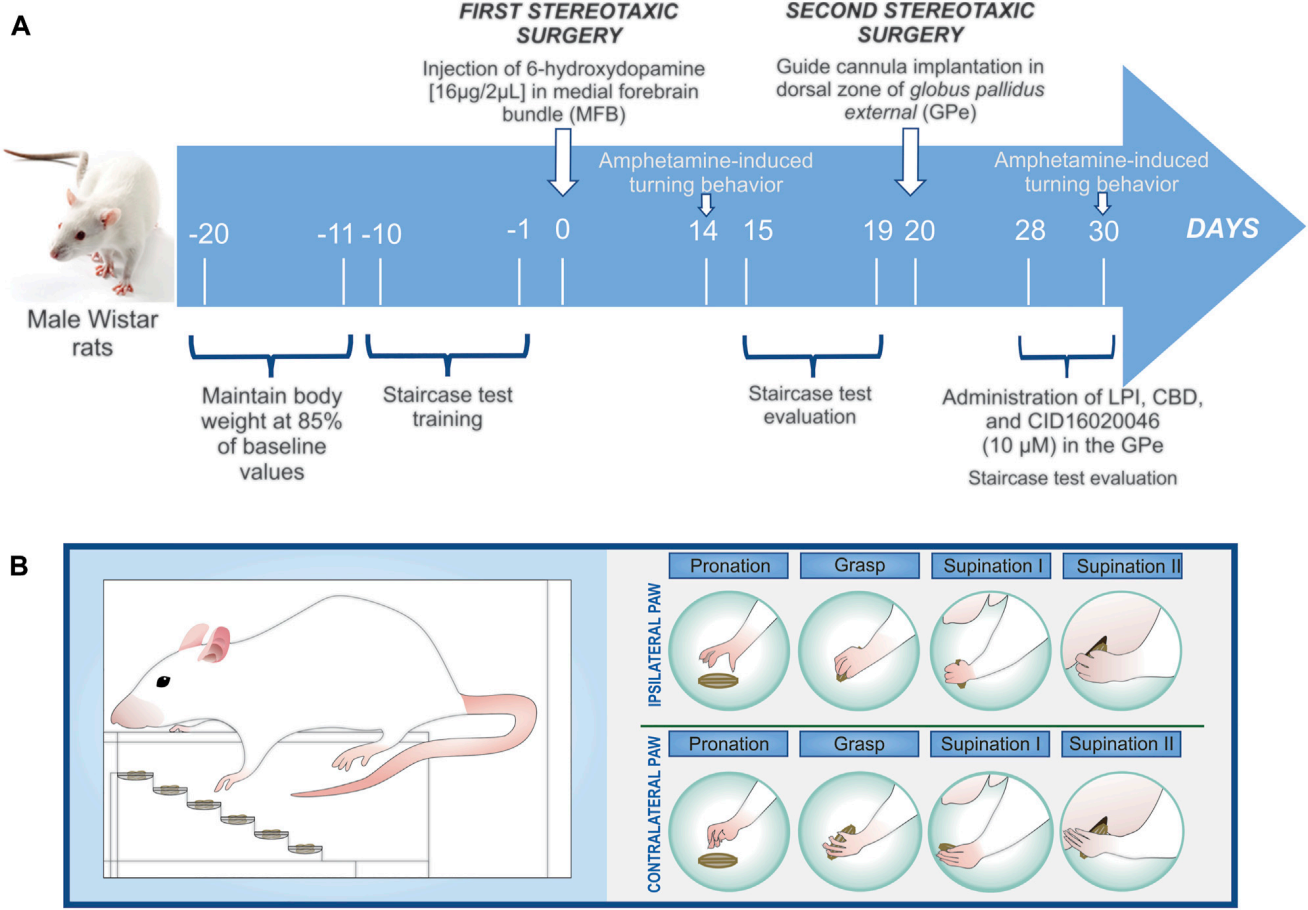
**(A)** Schematic of the experimental design. Day Zero is considered the beginning of the 6-OHDA-induced denervation of the dopaminergic neurons (the first stereotaxic surgery). Amphetamine-induced turning behavior was evaluated twice, on the 14th and 30th days post-lesion. Once the guide cannula had been implanted on the 15th day post-lesion (the second stereotaxic surgery), eight groups were obtained: vehicle + DMSO 0.01%; vehicle + LPI 10 μM; vehicle + CBD 10 μM; vehicle + CID160 10 μM; 6-OHDA + DMSO 0.01%; 6-OHDA + LPI 10 μM; 6-OHDA + CBD 10 μM; and, 6-OHDA + CID160 10 µM. The staircase test training was conducted ten days prior to the first stereotaxic surgery, with the motor alterations presenting in 6-OHDA-induced hemiparkinsonism then evaluated from the 15th to the 19th days post-lesion and the pharmacological effects of the drugs of interest then analyzed from the 28th to 30th days post-lesion. **(B)** The fine motor skills evaluated in the forelimbs ipsilateral and contralateral to lesion were pronation, grasp, supination I, and supination II.

Twenty days after the first surgical procedure (the 6-OHDA lesion administered in the MFB), a second procedure was undertaken to implant a guide cannula in the GPe, ipsilateral to the lesion (Figure 1). The cannula was used to locally administer the LPI [10 µM], CBD [10 µM], and CID16020046 [10 µM]. Once the rat’s skull had been exposed in the stereotaxic apparatus, it was trepanned and a 15-mm length of 22-gauge stainless steel tubing was then implanted in the top of the left GPe using the following coordinates: AP, -1.0 mm from the Bregma; ML, +2.9 mm from the midline; and, DV, -4.8 below the dura. Subsequently, the cannula was anchored to the skull with two stainless steel screws and secured with dental acrylic cement, with wire stylets then inserted into the guide cannula to prevent clogging. For all surgical procedures, postoperative care was applied, with an anti-inflammatory (12.5 mg/kg tramadol, s.c.) and antibiotic (10 mg/kg enrofloxacin, i.m.) administered, both on the day of surgery and the following two days, with the surgical wound checked every day until the animal made a full recovery.

### 2.3 Drugs

The following drugs were used in the present study: 4-[4,6-Dihydro-4-(3-hydroxyphenyl)-3-(4-methylphenyl)-6-oxopyrrolo[3,4-c] pyrazol-5(1H)-yl]-benzoic acid (CID16020046, Sigma-Aldrich, #SML0805); 6-hydroxydopamine (6-OHDA, Sigma-Aldrich, #H4381); ascorbic acid (Sigma-Aldrich, #A92902); d-Amphetamine (AMPH); dimethyl sulfoxide (DMSO, Sigma-Aldrich, #34869); l-α-lysophosphatidylinositol (LPI, Sigma-Aldrich, #L7635); and, cannabidiol (CBD), kindly provided by the company HempMeds^®^ México.

#### 2.3.1 Drug treatments

The GPR55 agonist LPI [10 µM], the GPR55 inverse agonist CBD [10 µM], the selective GPR55 antagonist CID16020046 [10 µM], and the vehicle dimethyl sulfoxide (DMSO) (0.01%) were injected using a 10 µL microsyringe (Hamilton digital syringe, Harvard Apparatus) and a 30-gauge, 16-mm-long stainless-steel internal cannula inserted into the guide cannula. The drugs were administered, *via* infusion at a volume of 1 µL for five minutes, for three consecutive days, namely the 28th to the 30th day post-6-OHDA-induced injury, in order to study the acute effect of the drugs on the GPe of rats subject freedom of movement. After a further five minutes, the animals were subjected to behavioral tests, which lasted a total of 10 min, thus allowing the drug to be distributed around the GPe.

### 2.4 Motor behavior tests

#### 2.4.1 Amphetamine-induced turning behavior

The experimental groups participating in the turning behavior test from the 28th day post-lesion onwards were as follows: Vehicle + DMSO 0.01% (*n* = 6); Vehicle + LPI 10 µM (*n* = 6); Vehicle + CBD 10 µM (*n* = 6); 6-OHDA + DMSO 0.01% (*n* = 6); 6-OHDA + LPI 10 µM (*n* = 6); and, 6-OHDA + CBD 10 µM (*n* = 8).

All experimental subjects were evaluated for amphetamine-induced turning behavior, in order to determine the degree of 6-OHDA-induced dopaminergic denervation in the MFB. Fourteen days post-6-OHDA injury or vehicle administration, each rat was administered with amphetamine (AMPH) (5 mg/kg s.c.) and then placed in a plastic box that was 50 cm in diameter and 40 cm long (Ramírez-García et al., 2015). The turning behavior exhibited by all the rats was monitored for up to 100 min and their turns quantified at 10-min intervals. Only those rats that presented 14 turns per minute were selected for the administration of the drugs of interest into the GPe. Subsequently, the intrapallidal administration of 1 µL of LPI [10 µM] and CBD [10 µM] was performed on the 28th, 29th, and 30th days post-dopaminergic injury, ten minutes after which, the AMPH (5 mg/kg s.c.) was administered, to enable the evaluation of turning behavior (Figure 1).

#### 2.4.2 Staircase test

The experimental groups participating in the staircase test from the 28th day post-lesion onwards were as follows: Vehicle + DMSO 0.01% (*n* = 6); Vehicle + LPI 10 µM (*n* = 5); Vehicle + CBD 10 µM (*n* = 5); Vehicle + CID160 10 µM (*n* = 5); 6-OHDA + DMSO 0.01% (*n* = 7); 6-OHDA + LPI 10 µM (*n* = 7); 6-OHDA + CBD 10 µM (*n* = 7); and, 6-OHDA + CID160 10 µM (*n* = 7).

##### 2.4.2.1 Apparatus

The staircase test, as proposed by Montoya et al. (1991), was used to measure the reaching and grasping abilities of the rats’ independent forelimb. This test can be used to evaluate motor asymmetry, in order to individually determine the grasping performance of both the unaffected (ipsilateral) and impaired (contralateral) paw of the hemiparkinsonian rats (Barnéoud et al., 2000; Klein et al., 2007; Cordeiro et al., 2010; Rattka et al., 2016). The apparatus used was in accordance with that described in previous studies (Mendieta et al., 2009) and consisting of a clear acrylic box (200 mm long, 100 mm wide, and 100 mm high) which channeled the rats to a narrower compartment (170 mm long, 65 mm wide, and 100 mm high) with a central platform running along its length. A removable double staircase comprising six steps of increasing distance from the top of the platform was placed in such a way that in ran from the front of the box into the troughs on both sides.

##### 2.4.2.2 Testing procedure

Two 45-mg pellets (Dustless Precision Pellets, #F0165, Bio-Serv Inc., United States) were placed on each step, which was 3 mm in height (Figure 1B), meaning that 12 pellets were placed on each of the six steps comprising the right and left staircases, giving a total of 24 pellets for both staircases. Measures were taken to control the animals’ body weight for the twenty days prior to the first stereotaxic surgery, from which point on and after each training session, the rats were provided a measured and decreasing (from 20 to 14 g) ration of food per day, in order to ensure that they presented 85% of their original weight until the surgery was carried out. Feeding was only resumed, on an *ad libitum* basis, after the surgeries were performed, in order to assist in the post-operative recovery, with the body weight control measures then implemented again six days prior to the procedures conducted on the 15th and 28th days post-injury to assess motor performance (Figure 1). The animals were familiarized with the pellets by being presented with them on two consecutive days prior to the beginning of training. Ten days prior to the first stereotaxic surgery, each rat was trained in the staircase test, with one trial conducted per day. The rats remained on the staircase for ten minutes, with the total number of pellets eaten then recorded. The objective of the training was to habituate the rats to consuming the pellets placed on the steps of the staircase. The same test was used to evaluate both the hemiparkinsonian rats’ motor asymmetry, from the 15th to the 19th day post-injury, and the effect of the drugs administered on motor asymmetry, from the 28th to the 30th day post-injury.

##### 2.4.2.3 Motor asymmetry evaluation

The present study used a modified version of the model reported by Nikkhah et al. (Nikkhah et al., 1998) and Klein et al. (2007) for examining the behavior of rats in the staircase test. After each daily 10-min evaluation period had been completed and the rats’ performance in the staircase test had been recorded, the number of pellets eaten, the number of remaining pellets, and the number of dislodged pellets were also recorded, with the latter two parameters described as follows: 1) the number of remaining pellets, refers to those left on the step on which they had originally been placed; and, 2) the number of dislodged pellets refers to those dropped onto other steps or beyond the staircases and into other areas of the box. The first parameter can be more precisely described as follows, along with two more parameters calculated based on the data obtained for the foregoing three parameters: 1) The number of pellets eaten (12 - [number of remaining pellets + number of dislodged pellets]) expresses the ratio of the number of pellets eaten to the total number of pellets placed; 2) The number of pellets taken (12 - number of remaining pellets) is considered to be a good measure of general reaching activity and motivation; and, 3) Grasping success in % ([number of pellets eaten/number of pellets taken] × 100) shows the level of success of all attempts to grasp a pellet. All trials were videotaped with a Sony DCR-SR85 video camera.

##### 2.4.2.4 Fine motor skills evaluation

The hemiparkinsonian rats’ grasping ability was qualitatively evaluated in the staircase test, as were their fine motor skills by analyzing the individual components of reaching and grasping patterns observed in high-speed video recordings. The evaluation was scored according to the scale described by Metz and Whishaw (2000), with some modifications. Each reaching movement was divided into four movement components: pronation; grasp; supination I; and, supination II. The four components were subdivided into ten subcomponents, with each subcomponent scored as a normal movement (1 point), an abnormal movement (0.5 points), or no movement (0 points), with a higher score indicating superior reaching movement performance and a lower score indicating inferior performance.

### 2.5 Histological examination

Thirty days after the administration of the 6-OHDA lesion and on the third day of the intrapallidal administration of the drugs of interest, the rats were euthanized by means of pentobarbital overdose and transcardially perfused with 4% paraformaldehyde in 0.1 M phosphate-buffered saline (PBS, pH 7.4). The brains were post-fixed in the same solution for 24 h. Coronal sections of 50 μm-thickness were cut, using a vibratome (VT1000S, Leica, Germany).

#### 2.5.1 Nissl staining

In order to study the cytoarchitecture of the GPe and verify the correct placement of the cannula, Nissl staining was performed, wherein brain sections were rinsed with 0.1 M PBS, pH 7.4, mounted on gelatinized slides, and stained with a 0.1% cresyl violet solution for 10 min. The sections were rinsed in distilled water and dehydrated with ascending grades of alcohol, cleared with xylene, and coverslipped with Entellan Mounting Medium.

#### 2.5.2 Tyrosine hydroxylase and glutamic acid decarboxylase (GAD-67) immunohistochemistry

Bright-field immunohistochemistry for TH and GAD-67 was carried out on tissue sections taken from the striatum, Gpe and SNpc *via* the application of the previously-described free-floating, protocol (Apóstol Del Rosal et al., 2021; Patricio et al., 2022). The sections were washed three times in PBS-Triton X-100 at 0.2% for 10 min and then treated with 3% hydrogen peroxide (H_2_O_2_) and 10% methanol to quench endogenous peroxidase activity. Subsequently, the sections were incubated for 1 h in a blocking buffer (IgG-free 2% bovine serum albumin in PBS-Triton X-100 at 0.2%) and incubated for two nights at 4°C with the primary antibody anti-TH (Merck Millipore, #MAB5280, 1:1000) or anti-GAD-67 (Santa Cruz Biothecnology, # SC-390383, 1:50). The sections were rinsed and incubated with biotinylated goat anti-mouse secondary antibody (Vector Laboratories, #BA-9200, 1:200) for two hours, followed by incubation in streptavidin horseradish peroxidase conjugate (Thermo Fisher, #43–4323, 1:500) for 1.5 h at room temperature. Finally, the sections were rinsed in 0.1 M PBS, pH 7.4, and incubated in 0.05% of 3,3-diaminobenzidine (DAB) and 0.03% H_2_O_2_ to visualize the immunocomplexes. Finally, the tissue sections were mounted onto gelatinized-coated slides, dehydrated in an ascending series of alcohols, cleared in xylene, and coverslipped with Entellan Mounting Medium. Images of the slides were captured using a Leica ICC50 camera with a Leica DM750 optical microscope, which was set at 4 × objective and the same level of contrast and sharpness for each slide, while the images analysis was performed with the ImageJ free software. The color intensities were converted into grayscale in order to graph the percentage of the area that had been stained, while the measurements were conducted on four animals per group, with three slices taken per animal.

### 2.6 Statistical analysis

The data are presented as mean ± standard error of the mean (SEM), wherein the results for amphetamine-induced turning behavior and fine motor skills were analyzed using a two-way analysis of variance (ANOVA) followed by a Bonferroni post-test. The motor asymmetry assessed in the staircase test was analyzed by means of a one-way ANOVA followed by a Tukey post-test. Results *p* < 0.05 were considered statistically significant for both analyses conducted. The software GraphPad Prism version 5.0 (GraphPad Software, La Jolla, CA, United States) was used for all statistical analyses.

## 3 Results

### 3.1 Effect of LPI and CBD administration in the external globus pallidus on amphetamine-Induced turning behavior in hemiparkinsonian rats

**FIGURE 2:**
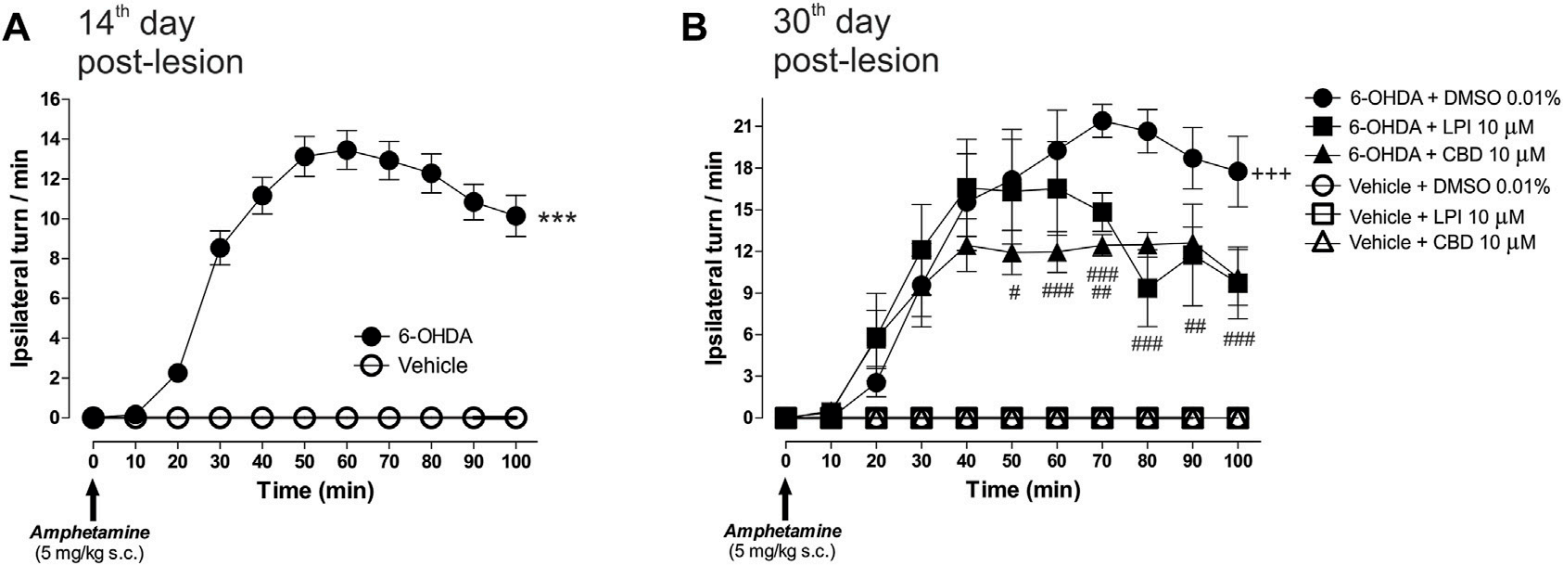
The administration of LPI and CBD in the GPe generated lower levels of 6-OHDA-induced motor asymmetry. **(A)** Amphetamine-induced turning behavior (5 mg/kg) was evaluated 14 days after the 6-OHDA injection into the MFB, with all animals placed into two groups–vehicle and 6-OHDA. The 6-OHDA group showed an increase in the number of ipsilateral turns per minute, in contrast to the vehicle group, which did not show any ipsilateral turns. **(B)** Six experimental groups were formed in order to evaluate the amphetamine-induced turning behavior presented by the animals after the intrapallidal administration of 1 µL [LPI 10 µM], [CBD 10 µM], or vehicle (DMSO 0.01%) on the 30th day post-lesion. Lower levels of 6-OHDA-induced motor asymmetry in 6-OHDA + LPI and 6-OHDA + CBD groups, a finding which contrasts with the 6-OHDA + DMSO. Data are represented as the mean ± SEM of ipsilateral turns per minute (10 periods of 10 min). ^***^ *p* < 0.001 *vs*. vehicle; ^+++^ *p* < 0.001 *vs*. vehicle + DMSO 0.01%; ^#^ *p* < 0.05, ^##^ *p* < 0.01 and ^###^ *p* < 0.001 *vs*. 6-OHDA + DMSO 0.01%; a two-way ANOVA followed by a Bonferroni *post-hoc* test, (*n* = 6–8/group).

The amphetamine-induced turning behavior was assessed on the 14th day post-injury, in order to examine the dopaminergic denervation in rats injected with 6-OHDA into the MFB. Figure 2A shows a higher number of ipsilateral turns in the 6-OHDA-lesioned group, with a maximum peak observed at Minute 60 (13.4 ± 0.9 turns/min) and a decrease by Minute 100 (10.1 ± 1.0 ipsilateral turns/min), which corresponds to a significant difference (*p* < 0.001) compared to the vehicle (ascorbic acid) group. Once the cannula had been implanted in the GPe, the rats were subdivided into six groups for the administration of the LPI [10 µM] and CBD [10 µM] on the 28th, 29th, and 30th days post-lesion, with the behavioral evaluation performed again on the 30th day. Figure 2B shows the turning behavior evaluated on the 30th day post-injury and the effect of the intrapallidal injection of both LPI and CBD. A significantly higher number of ipsilateral turns can be observed in the 6-OHDA + DMSO 0.01% group from Minute 30 onwards (9.5 ± 3.0 turns/min), reaching a maximum point at Minute 70 (21.4 ± 1.1 turns/min), and slightly decreasing by Minute 100 (17.7 ± 2.5 ipsilateral turns/min). Both the 6-OHDA + 10 µM LPI group and the 6-OHDA + 10 µM CBD group show an evident decrease in ipsilateral turns at the 70th and 80th minute intervals (14.8 ± 1.3 and 12.4 ± 0.7 ipsilateral turns/min; 9.3 ± 2.7 and 12.4 ± 0.8 ipsilateral turns/min), respectively (*p* < 0.01).

### 3.2 The administration of LPI in the external globus pallidus does not modify the motor asymmetry of hemiparkinsonian rats evaluated in the staircase test

**FIGURE 3:**
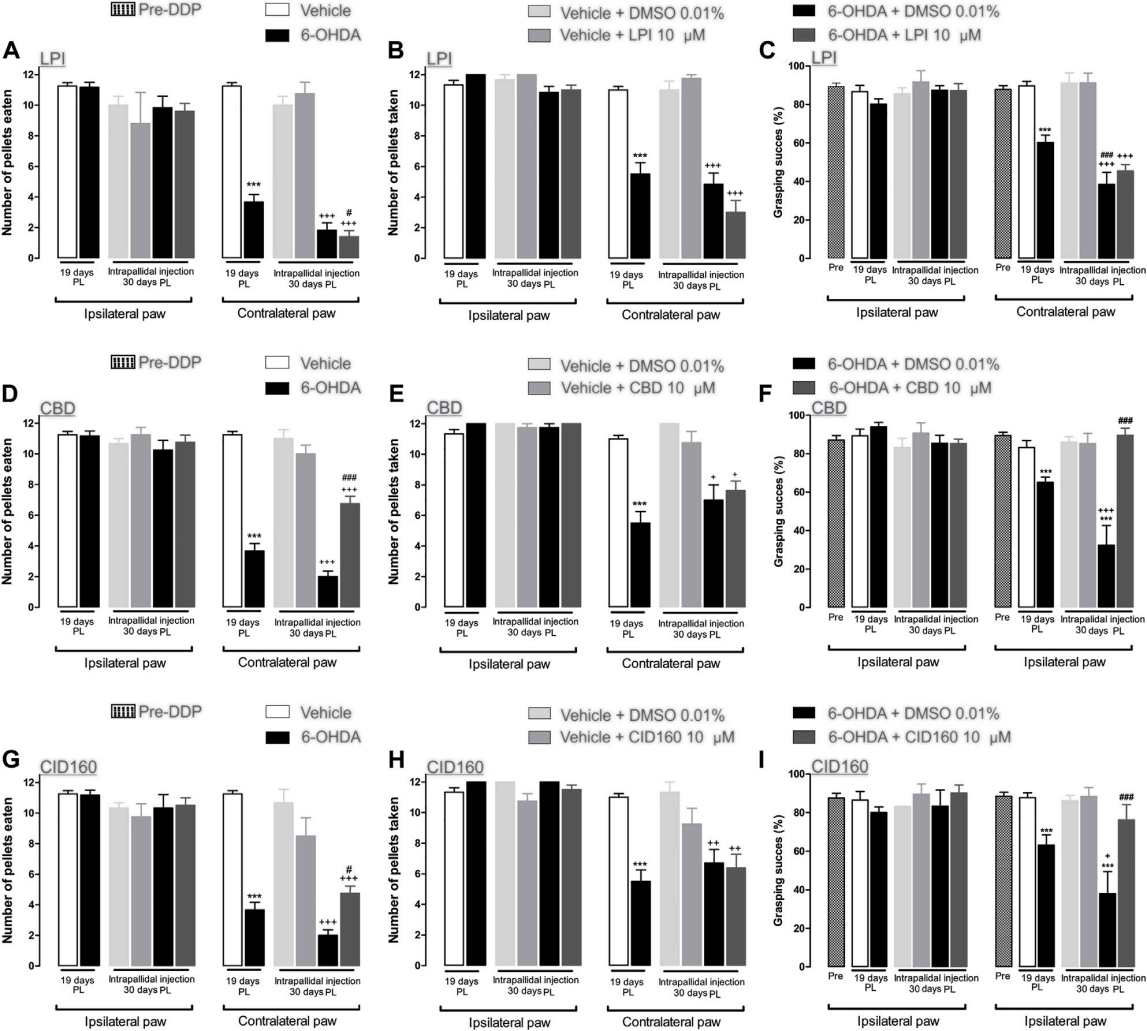
The CBD and CID160 administered in the GPe generated superior forelimb performance in the staircase test. The number of pellets eaten, the number of pellets taken, and the percentage of grasping success were recorded on the 19th day post-lesion. The GPR55r agonist LPI (10 µM) **(A–C)**, the inverse agonist CBD (10 µM) **(D–F)**, and the selective antagonist CID160 10 µM **(G–I)** were administered via intrapallidal injection on the 30th day. It is shown that the 6-OHDA + CBD and 6-OHDA + CID160 groups increase the indices of motor performance in contrast with the 6-OHDA + LPI and 6-OHDA + DMSO groups. Data are represented as group mean ± SEM. ^***^ *p* < 0.001 *vs*. vehicle; ^+^ *p* < 0.05, ^++^ *p* < 0.01, ^+++^ *p* < 0.001 *vs*. vehicle + DMSO 0.01%; ^#^ *p* < 0.05, ^###^ *p* < 0.001 *vs*. 6-OHDA + DMSO 0.01%; a one-way ANOVA followed by a Tukey *post-hoc* test, (*n* = 5–7/group).

The hemiparkinsonian model involved the unilateral injection of 6-OHDA into the MFB, thus impairing the BG circuit and inducing motor asymmetry (Taylor et al., 1992; Meredith et al., 2008; Glajch et al., 2012). Therefore, GPR55 was activated *via* the injection of the agonist LPI at a dose of [10 µM] *via* the guide cannula. On the 19th day post-lesion, the effect of the dopaminergic lesion on motor behavior was evaluated in the staircase model, with the results obtained showing a motor deficit in the limb contralateral to the lesion in the 6-OHDA group. This deficit is indicated by the number of pellets both eaten (3.6 ± 1.7) and taken (5.5 ± 0.74) and the percentage of grasping success (60.1 ± 3.9), when compared to the corresponding results obtained for the vehicle group (pellets eaten: 11.25 ± 0.21, pellets taken: 11.0 ± 0.23, and percentage of grasping success: 89.3 ± 2.6) (*p* < 0.001). On the 30th day post-lesion, the effect of each pharmacological treatment was evaluated. The 6-OHDA + DMSO and 6-OHDA + LPI groups presented a lower number of pellets eaten (1.8 ± 0.47; 1.4 ± 0.4) (Figure 3A) and taken (4.8 ± 0.73; 3.0 ± 0.77) (Figure 3B) and a lower percentage of grasping success (38.4 ± 6.2; 45.4 ± 3.3) (Figure 3C) than the vehicle + DMSO group (pellets eaten: 10.0 ± 0.57, pellets taken: 11.0 ± 0.57, percentage of grasping success: 91.1 ± 5.2). It should be noted that a statistically-significant difference was found between the percentage of grasping success observed for the 6-OHDA group on the 19th day post-lesion and that observed on the 30th day post-lesion (*p* < 0.001). Finally, the number of pellets eaten by the 6-OHDA + LPI group presented a statistically-significant difference to the 6-OHDA group on the 19th day post-injury. No significant changes were observed among the ipsilateral paw control groups (Figures 3A–C).

### 3.3 The administration of CBD and CID16020046 in the external globus pallidus decreases motor asymmetry in hemiparkinsonian rats evaluated in the staircase test

Cannabidiol was used to evaluate motor behavior in the staircase test. On the 19th day post-lesion, the effects of the dopaminergic injury were evaluated in the staircase model. The results obtained showed a motor deficit in the contralateral limb of the 6-OHDA group, as indicated by the lower number of pellets eaten (3.6 ± 0.4) and taken (5.5 ± 0.74) and the lower percentage of grasping success (83.1 ± 3.6) than those observed in the results obtained for the vehicle group (pellets eaten: 11.2 ± 0.75, pellets taken: 11.0 ± 0.70, percentage of grasping success: 89.3 ± 1.7). On the 30th day post-injury, the effect of each pharmacological treatment was evaluated. The 6-OHDA + DMSO groups showed a lower number of number of pellets eaten (2.0 ± 0.36) (Figure 3D) and taken (7.0 ± 1.0) (Figure 3E) and a lower percentage of grasping success (32.4 ± 10.1) (Figure 3F) than those observed for the vehicle + DMSO group (pellets eaten: 11.0 ± 0.57, pellets taken: 12.0 ± 0.01, percentage of grasping success: 86.0 ± 2.8). A statistically-significant difference was observed between the percentage of grasping success for the 6-OHDA group on the 19th day post-lesion and that observed for the 6-OHDA group by the 30th day post-lesion (*p* < 0.001). Unlike the 6-OHDA + DMSO control group, the 6-OHDA + CBD group presented a lower number of pellets eaten (6.7 ± 0.56) (Figure 3D) and taken (7.6 ± 0.62) (Figure 3E) than the vehicle + DMSO group (*p* < 0.001). Finally, the number of pellets eaten by the 6-OHDA + CBD group (6.7 ± 0.47) and the percentage of grasping success (89.5 ± 3.7) observed presented a significant difference to that observed for the 6-OHDA + DMSO group (*p* < 0.05 and *p* < 0.001, respectively) (Figure 3F). No significant changes were observed for the ipsilateral paw control groups.

The selective GPR55 antagonist was used to evaluate motor behavior in the staircase test. Figures 3G–I show the evaluation of the dopaminergic lesion in the staircase test on the 19th day after it was induced. The results obtained show a motor deficit for the contralateral limb of the 6-OHDA group, given that the number of pellets eaten (3.6 ± 1.7) and taken (12 ± 0.7) and the percentage of grasping success (63.1 ± 5.3) were lower than the corresponding results obtained for the vehicle group (pellets eaten: 11.2 ± 0.21, pellets taken: 11.0 ± 0.23, percentage of grasping success: 87.7 ± 2.5) (*p* < 0.001). The pharmacological effect of CID16020046 was evaluated on the 30th day post-lesion. The 6-OHDA + DMSO groups showed a lower number of pellets eaten (2.0 ± 0.36) (Figure 3G) and taken (6.7 ± 0.9) (Figure 3H) and a lower percentage of grasping success (37.9 ± 11.34) (Figure 3I) than the vehicle + DMSO group (pellets eaten: 10.6 ± 0.88, pellets taken: 11.3 ± 0.66, percentage of grasping success: 86.1 ± 2.8). A statistically-significant difference was found for the 6-OHDA group between the percentage of grasping success observed the 19th day post-lesion and that observed on the 30th day post-lesion (*p* < 0.001). Unlike the results observed for the 6-OHDA + DMSO control group, the 6-OHDA + CID16020046 group presented a lower number of pellets eaten (4.7 ± 0.47) (Figure 3G) and taken (6.3 ± 0.89) (Figure 3H) than the vehicle + DMSO group (*p* < 0.001). Finally, the number of pellets eaten by the 6-OHDA + CID16020046 group (4.7 ± 0.47) and the percentage of grasping success (76.1 ± 8.0) for the same group presented a significant difference to the results observed for the 6-OHDA + DMSO group (*p* < 0.05 and *p* < 0.001, respectively) (Figure 3I). No significant changes were observed for the ipsilateral paw control groups.

### 3.4 The intrapallidal injection of LPI dies not modify the fine motor skills of hemiparkinsonian rats

**FIGURE 4:**
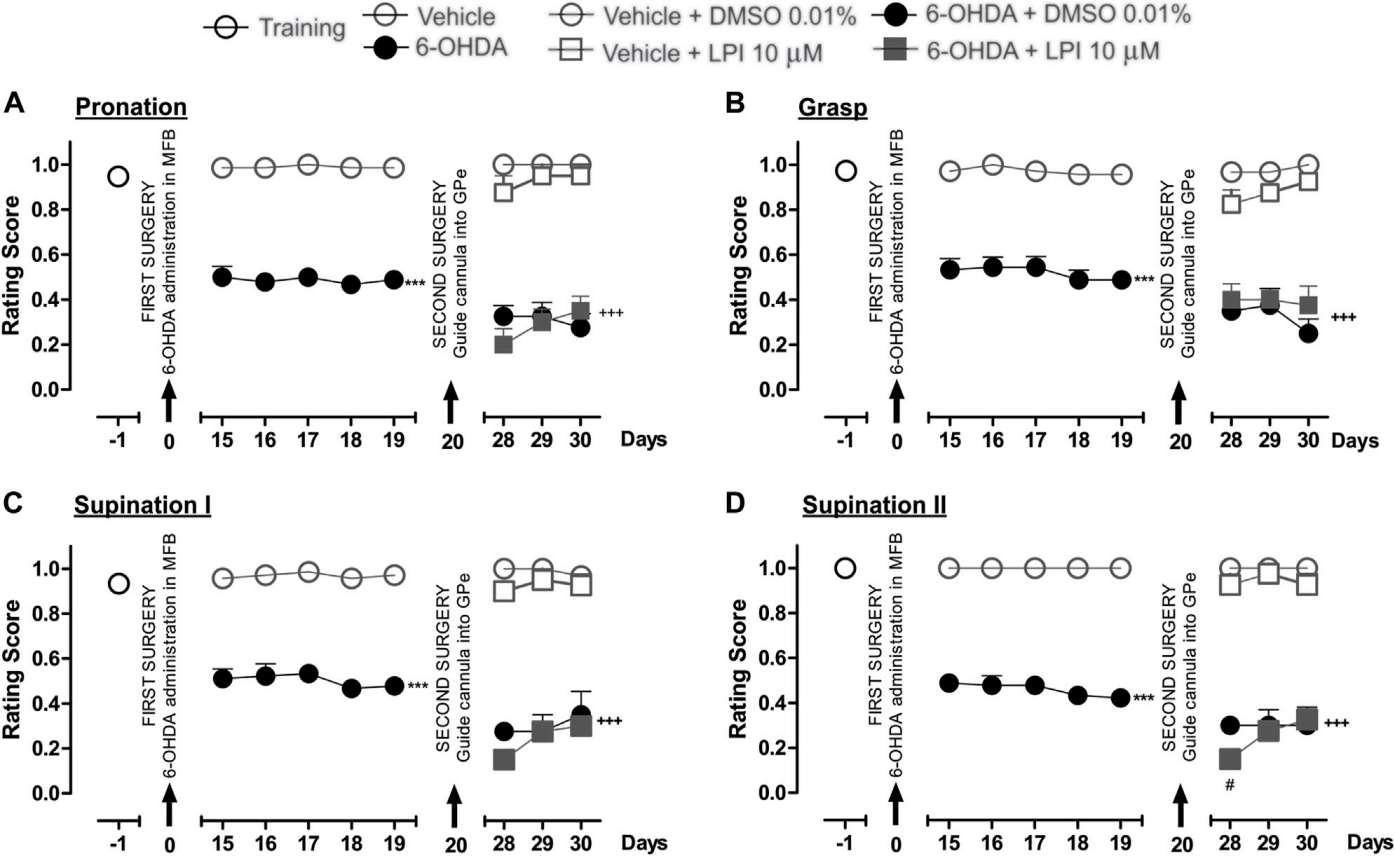
The administration of LPI in the GPe did not affect the fine motor skills of the hemiparkinsonian rats evaluated in the staircase test. The four movement components selected to evaluate the contralateral forelimb performance for fine motor skills were as follows: **(A)** Pronation; **(B)** Grasp; **(C)** Supination I; and, **(D)** Supination II. The effect of the intrapallidal administration of LPI (10 µM) on the movement components was evaluated one day prior to the first stereotaxic surgery (from the 15th to the 19th days post-lesion) and from the 28th to the 30th days post-lesion. It is shown that the 6-OHDA + LPI group performed worse in terms of fine motor skills than the control groups. Data are represented as the mean ± SEM of the fine motor skills scores obtained. ^***^ *p* < 0.001 *vs*. vehicle; ^+++^ *p* < 0.001 *vs*. vehicle + DMSO 0.01%; ^#^ *p* < 0.05 *vs*. 6-OHDA + DMSO 0.01%; a two-way ANOVA followed by a Bonferroni *post-hoc* test, (*n* = 5–7/group).

The performance of all the experimental groups evaluated in the staircase test was videotaped in order to later evaluate the rats’ fine motor skills. The video analysis conducted showed significantly lower scores for the pronation, grasp, supination I, and supination II movement components for the 6-OHDA group than those recorded for the control group (*p* < 0.001), as observed from the 15th to 19th day post-injury. After the intrapallidal administration of LPI, it was observed that the 6-OHDA + DMSO groups presented lower pronation scores, on the 28th, 29th, and 30th days post-injury, than the vehicle + DMSO group (0.3 ± 0.04; 0.3 ± 0.06; and, 0.2 ± 0.06, respectively). The 6-OHDA + LPI groups presented the following lower scores for the four movement components than the 6-OHDA + DMSO group (*p* < 0.001) on the 28th, 29th, and 30th days post-injury: pronation (Figure 4A) (0.2 ± 0.07; 0.3 ± 0.05; and, 0.3 ± 0.06, respectively); grasp (Figure 4B) (0.4 ± 0.07; 0.4 ± 0.04; and, 0.3 ± 0.08, respectively); supination I (Figure 4C) (0.1 ± 0.02; 0.2 ± 0.02; and, 0.3 ± 0.04, respectively); and, supination II (Figure 4D) (0.1 ± 0.02; 0.2 ± 0.02; and, 0.3 ± 0.04, respectively). The evaluation of the limb ipsilateral to the injury did not show statistically-significant changes for any of the movements evaluated during the course of the test.

### 3.5 The administration of CBD and CID16020046 in the external globus pallidus improves the fine motor skills of hemiparkinsonian rats

**FIGURE 5:**
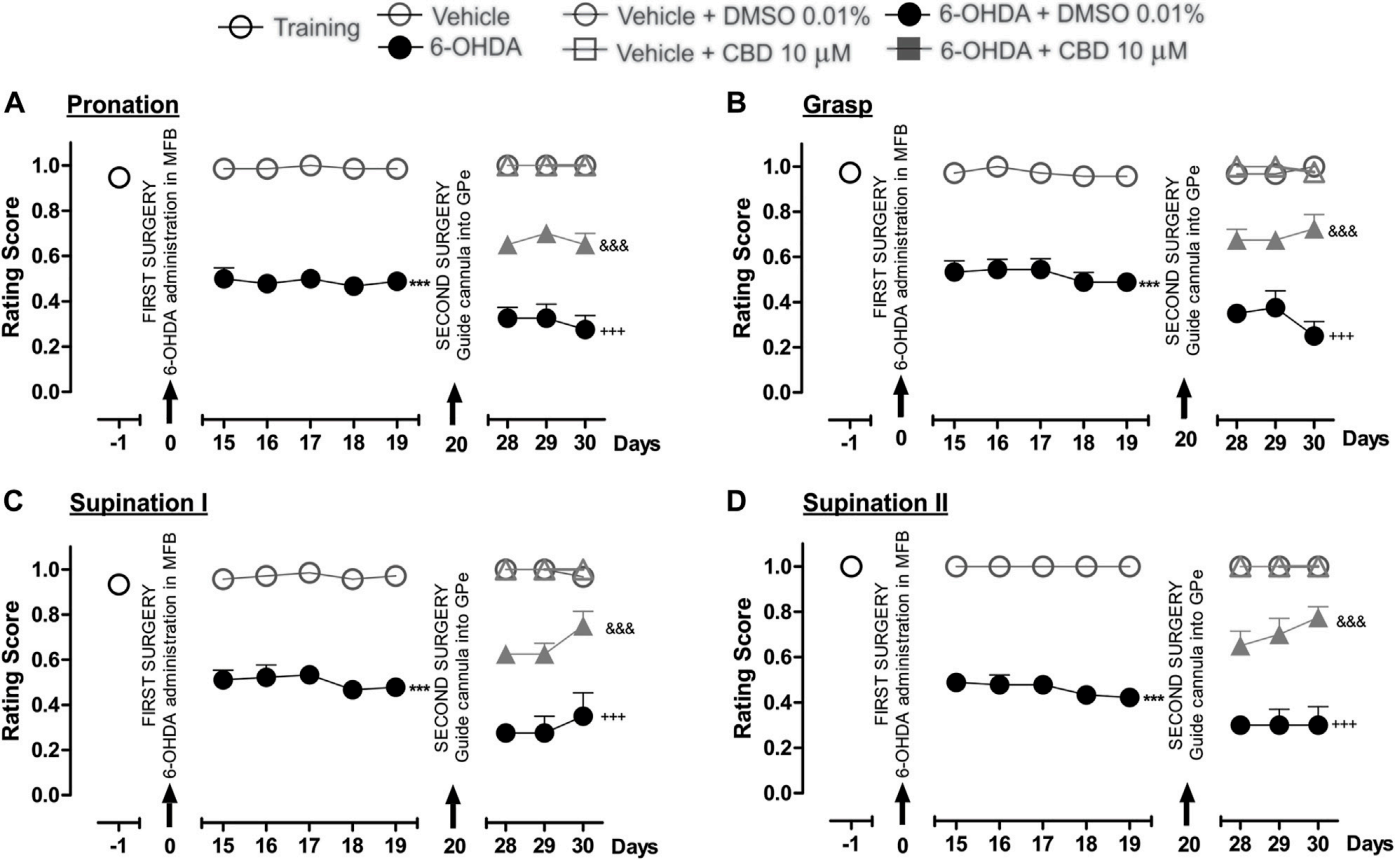
The administration of CBD in the GPe generated superior fine motor skills performance in the hemiparkinsonian rats analyzed in the staircase test. The four movement components selected to evaluate the contralateral forelimb performance for fine motor skills were as follows: **(A)** Pronation; **(B)** Grasp; **(C)** Supination I; and, **(D)** Supination II. The effect of the intrapallidal administration of CBD (10 µM) on the movement components were evaluated one day prior to the first stereotaxic surgery (from the 15th to the 19th days post-lesion) and from the 28th to the 30th days post-lesion. It is shown that 6-OHDA + CBD group performed better in terms of fine motor skills than the 6-OHDA + DMSO group. Data are represented as the mean ± SEM of the fine motors skills scores obtained. ^***^ *p* < 0.001 *vs*. vehicle; ^+++^ *p* < 0.001 *vs*. vehicle + DMSO 0.01%; ^&&&^ *p* < 0.001 *vs*. 6-OHDA + DMSO 0.01%; a two-way ANOVA followed by a Bonferroni *post-hoc* test (*n* = 5–7/group).

Figure 5 shows the effect of the intrapallidal injection of CBD on 6-OHDA-induced impaired fine motor skills. The hemiparkinsonian model showed significantly lower scores for the pronation, grasp, supination I, and supination II movement components for the 6-OHDA group than those observed for the control group (*p* < 0.001), from the 15th to the 19th day post-injury. After the intrapallidal administration of LPI, it was observed that the 6-OHDA + DMSO group presented lower scores than the vehicle + DMSO group for the 28th, 29th, and 30th days post-injury, for the following movement components: pronation (0.3 ± 0.04; 0.3 ± 0.06; and, 0.2 ± 0.05, respectively); grasp (0.3 ± 0.04; 0.2 ± 0.07; and, 0.2 ± 0.06, respectively); supination I (0.2 ± 0.02; 0.2 ± 0.07; and, 0.3 ± 0.1, respectively); and, supination II (0.3 ± 0.03; 0.3 ± 0.07; and, 0.3 ± 0.8, respectively). The 6-OHDA + CBD groups presented the following lower scores for the four fine movement components than the 6-OHDA + DMSO group (*p* < 0.001), for the 28th, 29th, and 30th days post-injury: pronation (Figure 5A) (0.6 ± 0.02; 0.7 ± 0.04; and, 0.6 ± 0.05, respectively); grasp (Figure 5B) (0.6 ± 0.04; 0.6 ± 0.02; and, 0.7 ± 0.06, respectively); supination I (Figure 5C) (0.6 ± 0.02; 0.6 ± 0.04; and, 0.7 ± 0.06, respectively); and, supination II (Figure 5D) (0.6 ± 0.06; 0.7 ± 0.07; and, 0.7 ± 0.04, respectively). The evaluation of the limb ipsilateral to the injury did not show statistically-significant changes for any of the movements evaluated during the course of the test.

**FIGURE 6:**
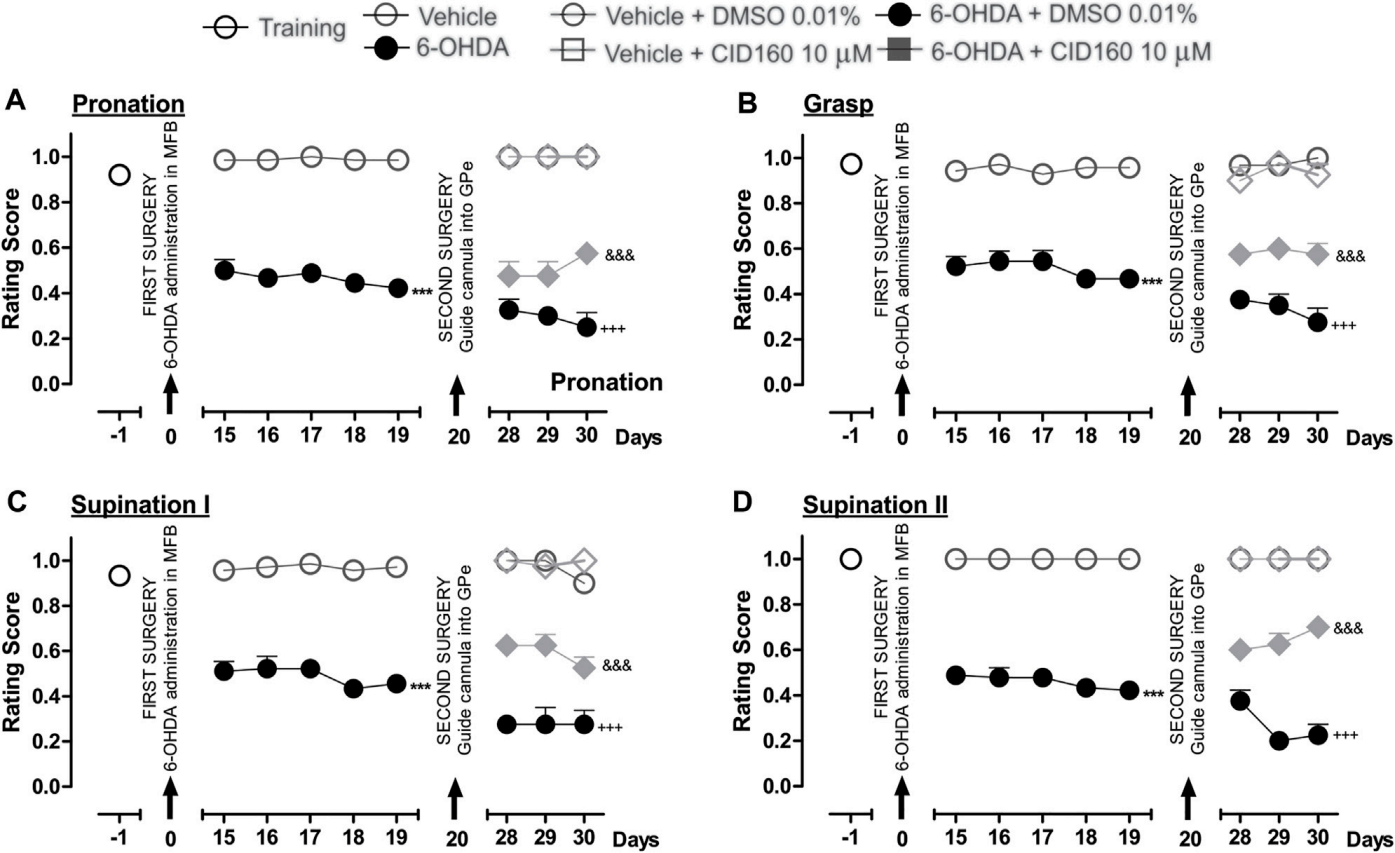
The administration of CID16020046 in the GPe generated superior fine motor skills performance in the hemiparkinsonian rats analyzed in the staircase test. The four movement components selected to evaluate the contralateral forelimb performance for fine motor skills were as follows: **(A)** Pronation; **(B)** Grasp; **(C)** Supination I; and, **(D)** Supination II. The effect of the intrapallidal administration of CID160 (10 µM) on the movement components were evaluated one day prior to the first stereotaxic surgery (from the 15th to the 19th days post-lesion) and from the 28th to the 30th days post-lesion. It is shown that 6-OHDA + CID160 group performed better in terms of fine motor skills than the 6-OHDA + DMSO group. Data are represented as the mean ± SEM of the fine motor skills scores obtained. ^***^ *p* < 0.001 *vs*. vehicle; ^+++^ *p* < 0.001 *vs*. vehicle + DMSO 0.01%; ^&&&^ *p* < 0.001 *vs*. 6-OHDA + DMSO 0.01%; a two-way ANOVA followed by a Bonferroni *post-hoc* test (*n* = 5–7/group).

Finally, Figure 6 shows the effect of the intrapallidal injection of CID16020046 on 6-OHDA-induced impaired fine motor skills. The hemiparkinsonian model showed significantly lower scores for the pronation, grasp, supination I, and supination II movement components for the 6-OHDA group than those observed for the control group (*p* < 0.001), from the 15th to the 19th day post-injury. After the intrapallidal administration of CID16020046, it was observed that the 6-OHDA + DMSO groups presented lower scores for the 28th, 29th, and 30th days post-injury than the vehicle + DMSO group for the following movement components: pronation (0.3 ± 0.04; 0.3 ± 0.04; and, 0.2 ± 0.06, respectively); grasp (0.3 ± 0.02; 0.3 ± 0.05; and, 0.2 ± 0.06, respectively); supination I (0.2 ± 0.02; 0.2 ± 0.07; and, 0.2 ± 0.06, respectively); and, supination II (0.3 ± 0.04; 0.2 ± 0.04; and, 0.2 ± 0.04, respectively). The 6-OHDA + CID16020046 groups presented lower scores for the following four fine movement components than the 6-OHDA + DMSO group (*p* < 0.001) for the 28th, 29th, and 30th days post-injury: pronation (Figure 6A) (0.4 ± 0.06; 0.4 ± 0.06; and, 0.5 ± 0.02, respectively); grasp (Figure 6B) (0.5 ± 0.02; 0.6 ± 0.04; and, 0.5 ± 0.04, respectively); supination I (Figure 6C) (0.6 ± 0.02; 0.6 ± 0.04; and, 0.5 ± 0.04, respectively); and, supination II (Figure 6D) (0.6 ± 0.06; 0.6 ± 0.06; and, 0.7 ± 0.07, respectively). The evaluation of the limb ipsilateral to the injury did not show statistically significant changes for any of the movement components evaluated during the course of the test.

### 3.6 The administration of LPI, CBD, and CID16020046 does not modify the expression of tyrosine hydroxylase in the striatum and substantia nigra pars compacta or the cell morphology in the external globus pallidus of hemiparkinsonian rats

**FIGURE 7:**
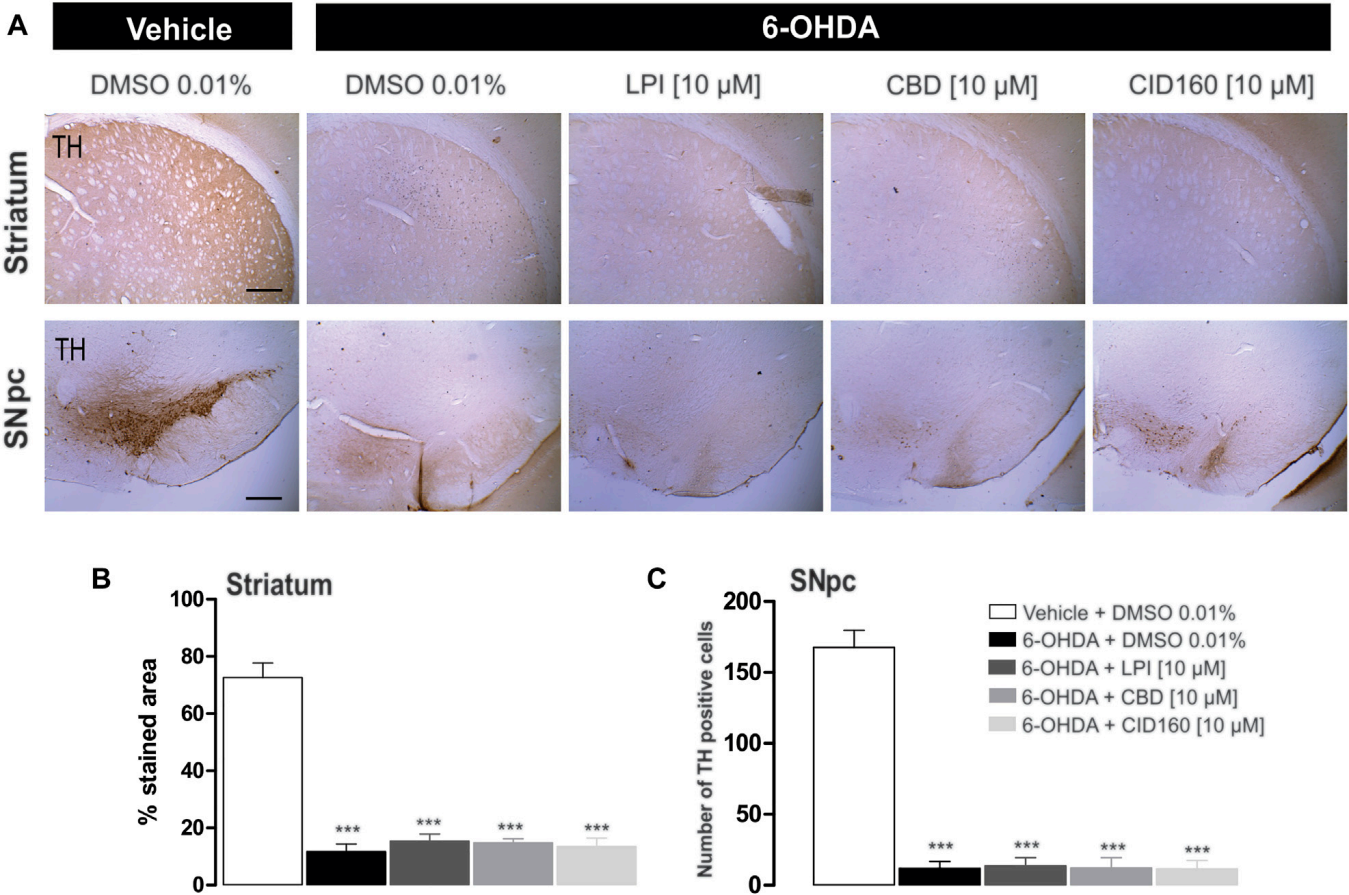
The administration of LPI, CBD, and CID16020046 does not modify the expression of TH in the striatum and SNpc. The photomicrography in **(A)** shows the representative images for TH expression in the striatum ipsilateral to the 6-OHDA lesion induced in the MFB. Photomicrographs were taken at 40x, while the scale bar represents 500 µm. Graph **(B)** shows the proportion of the area stained for TH in the striatum and Graph **(C)** shows the number of TH-positive cells in the SNpc. All groups show decreased immunoreactivity to the TH enzyme. The values show mean ± SEM (*n* = 4 per group). The statistics were determined *via* a one-way ANOVA and a Tukey *post-hoc* test. ^***^ *p* < 0.001 *vs*. vehicle group.

**FIGURE 8:**
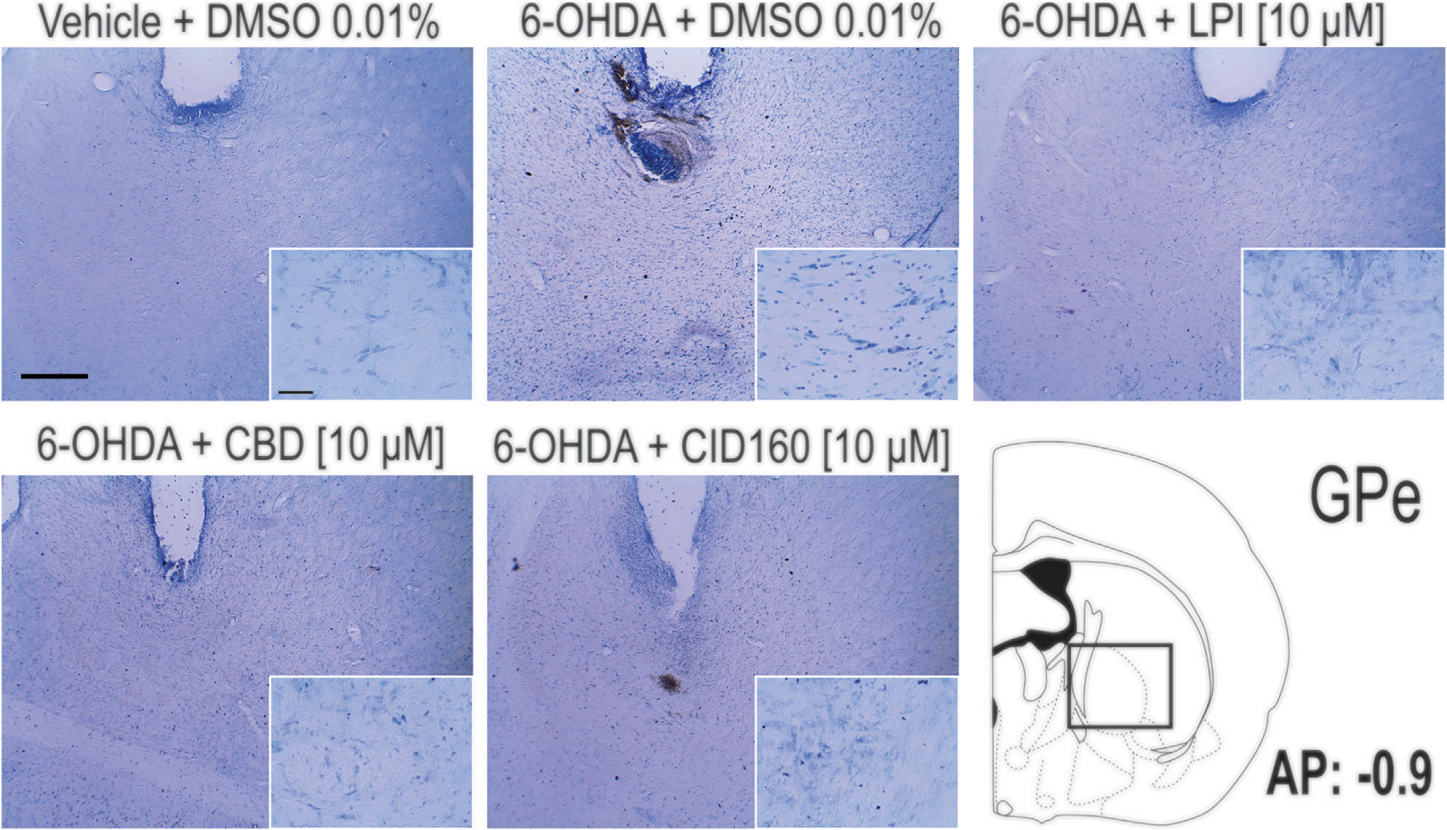
Pallidal cell morphology and verification of the correct placement of the cannula in the nucleus of the GPe. The photomicrographs show the images obtained *via* the Nissl staining of the GPe and the placement of the cannula in the dorsal area of the GPe. Photomicrographs were taken at 4x and 40x and the scale bar represents 500 and 50 μm, respectively.

In order to corroborate the success of the 6-OHDA-induced dopaminergic injury, TH immunoreactivity was performed in both the striatum and the SNpc (Figure 7). The graphs presented in Figures 7B,C show that the 6-OHDA + DMSO group presented a lower percentage area in the striatum stained for the TH enzyme (11.6 ± 2.6 percentage stained area) and a lower number of TH positive cells in the SNpc (13.2 ± 5.0 number of TH positive cells) (Figure 7C) than the control group (72.6 ± 5.0 percentage stained area) (167.6 ± 12.1 number of TH positive cells), with a significance of *p* < 0.001. No significant differences were found among the 6-OHDA + LPI, 6-OHDA + CBD, 6-OHDA + CID16020046 experimental groups for both the percentage area in the striatum stained for the TH enzyme (15.3 ± 2.4; 14.7 ± 1.4; 13.5 ± 2.8, respectively) and the number of TH positive cells in the SNpc (16.2 ± 6.0; 14.4 ± 5.4; 13.2 ± 4.7, respectively). The foregoing findings similarity with the results obtained for the 6-OHDA + DMSO group. Nissl staining was undertaken to reveal the cytoarchitecture and verify the correct placement of the cannula in the nucleus of the GPe. Figure 8 shows the placement of the cannula in the dorsal area of the GPe.

### 3.7 The intrapallidal injection of CBD or CID16020046 decreases GAD67 expression in the external globus pallidus and striatum of hemiparkinsonian rats

**FIGURE 9:**
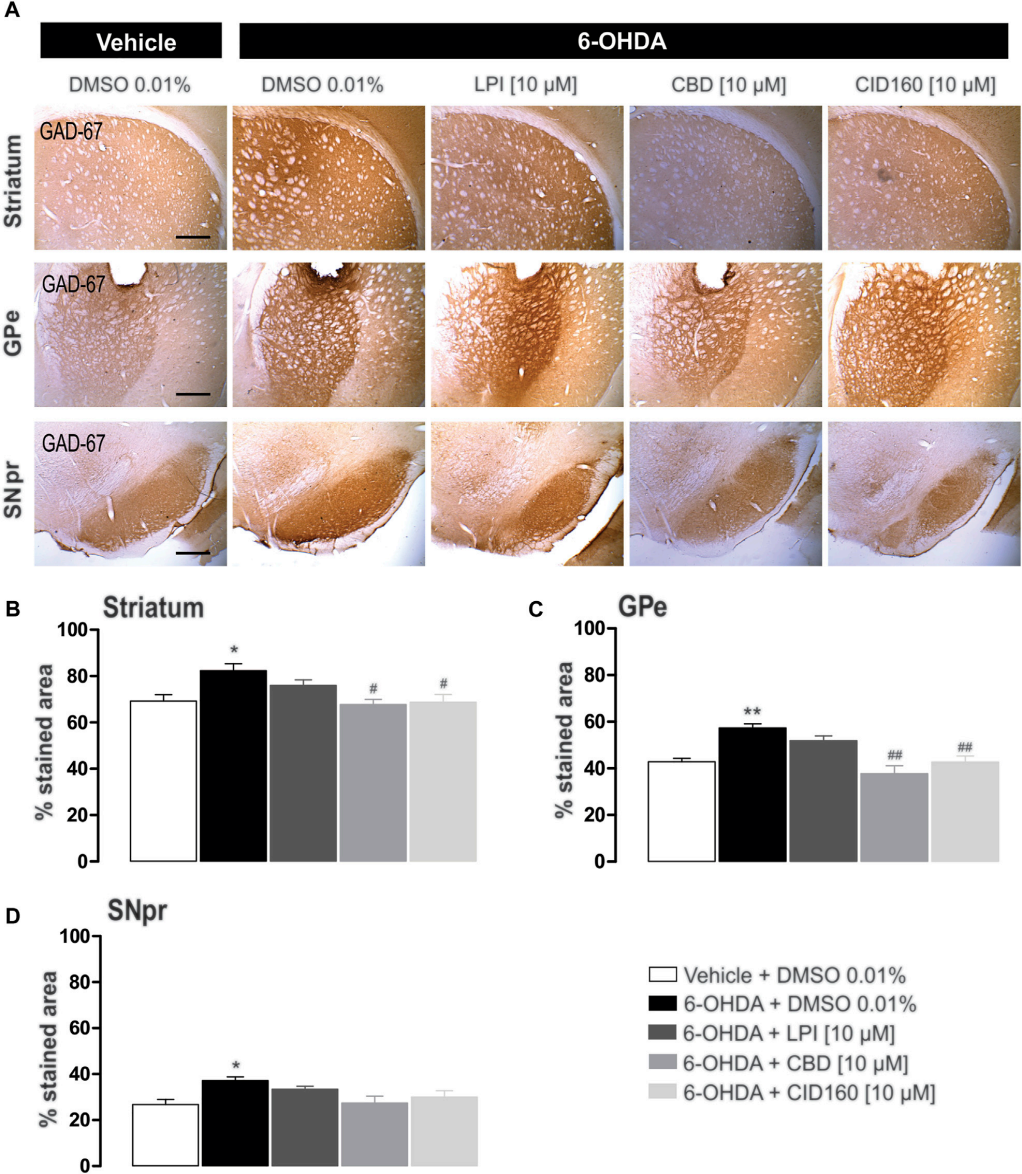
The intrapallidal injection of CBD or CID16020046 decreases the immunoreactivity of GAD-67 in the GPe and striatum of rats with 6-OHDA-induced injury. **(A)** shows the immunoreactivity of GAD-67 in the striatum, GPe, and SNpr. Photomicrographs were taken at 40x, while the scale bar represents 500 µm. Graph **(B)** shows the proportion of the area stained for TH in the striatum, while **(C,D)** show the percentage area stained for TH in the GPe and SNpr, respectively. The results obtained show that GAD-67 expression increased in the ipsilateral striatum, GPe, and SNpr of the 6-OHDA + DMSO group in contrast with the control group. However, a decreased level of GAD-67 immunoreactivity was only observed in the nuclei of the striatum and GPe ipsilateral to the lesion in 6-OHDA + CBD and 6-OHDA + CID160 groups. The values show mean ± SEM (*n* = 4 per group). Statistics were determined with a one-way ANOVA and a Tukey *post-hoc* test. ^*^ *p* < 0.05, ^**^ *p* < 0.01 *vs*. vehicle group. ^#^ *p* < 0.05, ^##^ *p* < 0.01 *vs*. 6-OHDA + DMSO group.

In order to explore the effect of the intrapallidal administration of LPI, CBD, or CID16020046 on 6-OHDA-induced alterations of GAD-67 expression, GAD-67 immunoreactivity in the GPe, striatum, and SNpr was measured (Figure 9A). The results obtained show that GAD-67 expression, in the striatum ipsilateral to the lesion (presented in Figure 9B) and the ipsilateral GPe, (presented in Figure 9C), increased significantly (*p* < 0.01 and *p* < 0.001, respectively) for the 6-OHDA + DMSO group (82.3 ± 2.9, 57.3 ± 1.7 percentage stained area) compared to the control group (69.2 ± 2.7, 42.8 ± 1.4 percentage stained area). Moreover, a lower level of GAD-67 immunoreactivity was observed for the groups treated with 6-OHDA + CBD and 6-OHDA + CID16020046 in the striatum (67.6 ± 2.2, 68.7 ± 3.3 percentage stained area) and GPe (37.7 ± 3.3, 42.7 ± 2.5 percentage stained area), with a significance of *p* < 0.01 and *p* < 0.001, respectively. The 6-OHDA + LPI group did not show differences to the 6-OHDA + DMSO group, in both the striatum (75.9 ± 2.3 percentage stained area) and GPe (51.8 ± 2.0 percentage stained area).

The GAD-67 expression observed in the SNpr was higher for the 6-OHDA + DMSO group (37.1 ± 1.7 percentage stained area) than the control group (26.8 ± 2.1 percentage stained area) (Figure 9D). No differences were observed among the 6-OHDA + LPI, 6-OHDA + CBD, 6-OHDA + CID16020046 experimental groups for the percentage area stained for the GAD-67 enzyme in the SNpr (33.5 ± 1.3; 27.4 ± 2.9; 30.0 ± 2.8, respectively), findings which contrast with the 6-OHDA + DMSO group.

## 4 Discussion

The present study evaluated the effect, on motor asymmetry and fine motor skills, of the intrapallidal administration of LPI, CBD, and CID16020046, which acted as an agonist, inverse agonist, and selective antagonist of GPR55, respectively, in a 6-OHDA-induced hemiparkinsonian model. Motor asymmetry was evaluated in amphetamine-induced turning behavior, while fine motor skills were evaluated in the staircase test. Immunoreactivity to the TH and GAD-67 enzymes in the nuclei of the striatum, GPe, and SNpc ipsilateral to the 6-OHDA-induced injury was also evaluated.

The results obtained show that the intrapallidal administration of LPI [10 µM] in hemiparkinsonian rats caused motor deficits in the staircase test and a deficit in fine motor skills, while a lower level of amphetamine-induced turning behavior was also observed (Figure 2B). The latter finding is of particular interest due to reported alterations in the BG circuit in parkinsonian states, especially those that presented increased GABAergic activity in the GPe (Galvan and Wichmann, 2008). Said reports show the expression of GPR55 mRNA in the BG (mainly in the GPe), the subthalamic nucleus (STN), and the striatum (Henstridge et al., 2011; Celorrio et al., 2017), proposing that the receptor plays an important role in GABA release. Therefore, the agonism of GPR55 to LPI would increase the release of GABA, as has been observed with other agonists such as palmitoylethanolamine (Musella et al., 2017). It is likely that the effects observed in terms of a lower incidence of turning behavior due to the intrapallidal administration of LPI are attributed to the activation of GPR55 in the afferents of other neuronal populations, such as the STN (Celorrio et al., 2017), which can be attributed to the findings reported for the nerve impulses received by the GPe from the striatum, STN, and SNpc (Eid and Parent, 2016). Furthermore, it has been shown that the STN presents efferents to the GPe (Kita and Kitai, 1987) and that the modulation occurring in both nuclei is strongly correlated in parkinsonian models (Kovaleski et al., 2020). The dopaminergic system does affect the GPe through the activation of the D1 receptor in the presynapse of the STN and the consequent release of glutamate (Hernández et al., 2007). Therefore, both the intrapallidal administration of LPI and the subcutaneous administration of amphetamine in hemiparkinsonian rats could modulate the GABAergic tone of the GPe as a function of the time period for which turning behavior is evaluated.

The intrapallidal administration of CBD [10 µM] conducted in the present study showed a more consistent and beneficial effect in terms of decreasing turning behavior in hemiparkinsonian rats. Cannabidiol is known to have different molecular targets, such as the CB1 receptor, the CB2 receptor, the fatty acid amide hydrolase (FAAH), the PPARγ receptor, the A2A receptor, the 5-HT1A receptor, and GPR55 (Peres et al., 2018). The effect of CBD as an inverse agonist of GPR55 has been widely accepted, as has its probable neuromodulatory role (Patricio et al., 2020). The negative effects of CBD on GPR55 in terms of glutamatergic, adenosinergic, and dopaminergic neurotransmission have been demonstrated *in vitro* (Ryberg et al., 2007; Sharir and Abood, 2010; Pandolfo et al., 2011). Therefore, the effect, as observed in the present study, exerted by CBD in reducing amphetamine-induced turning behavior should not only be interpreted in terms of its interaction with GPR55. In addition, the expression and activity of the A2A (Diao et al., 2017), 5-HT (Parent et al., 2011), and CB1 receptors have been demonstrated in the GPe (Chaves-Kirsten et al., 2013).

The evaluation of motor asymmetry in the staircase model was performed in the present study using the following parameters: number of pellets eaten; number of pellets taken; and, the percentage of grasping success. The staircase model enables the grasp capacity of the limb contralateral to the injury to be analyzed. Therefore, the results can be interpreted in terms of these three parameters: 1) The motivation and action required to attain a goal (number of pellets taken); 2) The ability to reach and grasp (number of pellets eaten); and, 3) The success of the attempts to grasp a pellet (grasping success percentage) (Klein et al., 2007). It was observed that the injection of LPI into the GPe of hemiparkinsonian rats caused a motor deficit in the limb contralateral to the injury, as observed in the staircase test (Figure 3), while motivation, grasping ability, and grasping success decreased. This finding is contrary to that observed in the tests conducted on turning behavior, due to the way in which the agonism of GPR55 to LPI in the GPe decreased the number of turns ipsilalateral to the lesion, a difference in behavior that is likely due to the dopamine released with the administration of amphetamine. The present study has also shown that the lower scores for fine motor skills presented by the groups injured with 6-OHDA and those in receipt of the intrapallidal administration of LPI on the 28th, 29th, and 30th days post-lesion (Figures 5, 6) are likely due to the low concentrations of bioavailable dopamine in the nigrostriatal and nigropallidal pathways (Rajput et al., 2008). Therefore, the absence of dopamine would cause a decrease in D2 receptor signaling and would also lead to an increase in striatopallidal and nigropallidal GABAergic tone. This absence could lead to the neuronal repolarization of the pallidosubthalamic pathway and may be translated into the over activation of glutamatergic neurons moving towards the BG output nuclei, thus promoting hypokinesia (Galvan and Wichmann, 2008; Lanciego et al., 2012).

The GPR55 receptor can activate the ROCK and/or PLC signaling pathway and induce the intracellular release of Ca^2+^ in the GABAergic neurons of the GPe. As this mechanism has only been reported in the hippocampal (Sylantyev et al., 2013) and dorsal root ganglia neurons (Lauckner et al., 2008), it is proposed that it also occurs in the hippocampal GABAergic neurons *via* the activation of GPR55 (Musella et al., 2017). The consequent release of intracellular Ca^2+^ in GABAergic neurons would trigger neuronal depolarization, which could result in an inhibition of GABA release and a decrease in hypokinesia. Our findings at the pharmacological level suggest that the activation of GPR55 in the GPe does not generate a change in the motor deficit observed in hemiparkinsonian rats, with both an increase in motor asymmetry and a decrease in fine motor skills observed. It is probable that this process is related to increased levels of GABA in the striatopallidal pathway (Galvan et al., 2005) which, acting alongside the antagonism, may reverse the overactivation. Thus, it is proposed that the indirect pathway of the GB circuit participates in the inhibition of movements corresponding to fine motor skills.

The intrapallidal injection of both CBD and CID16020046 led to a reduction in the motor asymmetry of hemiparkinsonian rats and an improvement in the fine motor skills of the contralateral limb, in contrast to the results obtained for the 6-OHDA + DMSO group. Interestingly, while there were no differences in the number of pellets taken, a parameter related to general reaching activity and motivation, the number of pellets eaten did increase, as did the grasping success percentage, which is a parameter related to the balance between motivation and grasping ability. These data provide evidence to support the proposal that GPR55 antagonism improves grip quality in hemiparkinsonian rats and may be related to neurotransmitter release (Marichal-Cancino et al., 2017). The results obtained reveal that CBD increased the grasping success percentage to a similar degree to that observed for the control groups. This finding may implicate the modulatory mechanism of CBD, as, after 30 days of 6-OHDA-induced injury, a higher percentage of dopaminergic neurons in the nigrostriatal pathway have degenerated and the neuroprotective role of CBD has been reduced to nil (García-Arencibia et al., 2007). The findings of the present study regarding motor asymmetry and fine motor skills provide evidence of the probable effect exerted on fine motor skills by GPR55 in the GPe of hemiparkinsonian rats.

## 5 Conclusion

In conclusion, these results provide findings pointing to the possible role played by CBD in the GPe of hemiparkinsonian rats, in terms of both gross and fine motor skills. Moreover, the effect on motor behavior of the selective GPR55 antagonist CID16020046 is similar to that obtained *via* CBD, thus opening new perspectives for explaining, at a cellular level, the role played by GPR55 in the GABAergic system and the reversal of the motor impairment observed in PD models.

## Data Availability

The original contributions presented in the study are included in the article/Supplementary Material, further inquiries can be directed to the corresponding author.

## Abbreviations

AMPH, Amphetamine; BG, Basal ganglia; CB_1,_ Cannabinoid receptor type 1; CBD, Cannabidiol; CNS, Central nervous system; DMSO, Dimethyl sulfoxide; ER, Endoplasmic reticulum; FAAH, Fatty acid amide hydrolase; GAD-67, Glutamic acid decarboxylase 67; GPe, external globus pallidus; GPR55, G-protein coupled receptor 55; H_2_O_2_, Hydrogen peroxide; 6-OHDA, 6-hydroxydopamine; LPI, Lysophosphatidylinositol; MPP+, 1-methyl-4-phenylpyridinium; MPTP, 1-methyl-4-phenyl-1,2,3,6-tetrahydropyridine; PD, Parkinson’s disease; PLC, Phospholipase C; ROCK, Rho-associated protein kinase; SNpc, substantia nigra pars compacta; SNpr, substantia nigra pars reticulata; STN, Subthalamic nucleus; TH, Tyrosine hydroxylase; THC, Δ^9^-tetrahydrocannabinol.
